# Relations between mental health team characteristics and work role performance

**DOI:** 10.1371/journal.pone.0185451

**Published:** 2017-10-09

**Authors:** Marie-Josée Fleury, Guy Grenier, Jean-Marie Bamvita, Lambert Farand

**Affiliations:** 1 Department of Psychiatry, McGill University, Montreal, Quebec, Canada; 2 Douglas Mental Health University Institute Research Centre, Montreal, Quebec, Canada; 3 Department of Health Administration, Policy and Evaluation School of Public Health, University of Montreal, Montreal, Quebec, Canada; UNITED STATES

## Abstract

Effective mental health care requires a high performing, interprofessional team. Among 79 mental health teams in Quebec (Canada), this exploratory study aims to 1) determine the association between work role performance and a wide range of variables related to team effectiveness according to the literature, and to 2) using structural equation modelling, assess the covariance between each of these variables as well as the correlation with other exogenous variables. Work role performance was measured with an adapted version of a work role questionnaire. Various independent variables including team manager characteristics, user characteristics, team profiles, clinical activities, organizational culture, network integration strategies and frequency/satisfaction of interactions with other teams or services were analyzed under the structural equation model. The later provided a good fit with the data. Frequent use of standardized procedures and evaluation tools (e.g. screening and assessment tools for mental health disorders) and team manager seniority exerted the most direct effect on work role performance. While network integration strategies had little effect on work role performance, there was a high covariance between this variable and those directly affecting work role performance among mental health teams. The results suggest that the mental healthcare system should apply standardized procedures and evaluation tools and, to a lesser extent, clinical approaches to improve work role performance in mental health teams. Overall, a more systematic implementation of network integration strategies may contribute to improved work role performance in mental health care.

## Introduction

Over the last two decades, most industrial countries have reformed their mental healthcare system[1–3]. Although there may be some differences regarding targeted mental disorders, structure, or clinical interventions, these reforms pursue similar objectives: a better response to the needs of persons with mental health disorders through enhanced accessibility, quality and continuity of care. Mental health reforms seek to widen access to care by prioritizing mental health services in primary care [4], which are less stigmatizing than specialized services. Concerning quality of care, the reforms promote evidence-based practices (e.g. strength model [5] for severe mental disorders, and stepped care [6] for depressive and anxiety disorders); these reduce mental health symptoms and increase quality of life for users with mental health disorders [7]. Finally, reforms favor a better integration of primary care and mental health services, through implementation of various clinical and administrative strategies, as a way to improve continuity of treatment and patient follow-up [8, 9]. Several studies concerning collaboration between primary care and specialized services for users with depression found that integrated care reduces depression symptoms, improves quality of life, and increases treatment compliance [10, 11]. Collaborative care between primary and specialized services also enhances quality of care for users with severe mental or substance abuse disorders [12].

Additionally, mental health disorders have biopsychosocial causes and—especially in the case of severe mental health disorders—affect both health and non-health aspects of life [13]. Their treatment requires therefore that professionals including psychiatrists, psychologists, nurses, social workers, educators and general practitioners work together within specialized and primary care services. Multidisciplinary teams working in several health areas provide more benefits to users than traditional care by a single professional [14]. According to the literature [15–17], more effective teams generate better treatment outcomes and greater satisfaction among users. Effective teamwork also reduces health costs [18] and medical errors [19] and contributes to job satisfaction among staff [15, 16].

While several factors may account for team effectiveness, work role performance is of major importance. Work role performance and team effectiveness are two distinct but closely related concepts. Effectiveness has been described as the capacity of teams to achieve desired results [20]. Work role performance refers to behaviors and actions of team members [21] that enhance effectiveness at individual, team and organizational levels [22]. Work role performance is thus an antecedent of effectiveness [22]. According to Griffin, Neal & Parker [22], work role performance includes proficiency, adaptivity, and proactivity. Proficiency is the extent to which members meet role requirements [22]; adaptivity is the capacity to cope with changes affecting their role [22]; and proactivity the capacity to foresee changes in working methods or procedures before problems occur [23]. Each sub-dimension is essential for team effectiveness, particularly given the nature of multidisciplinary teamwork involving high levels of uncertainty in treating users with severe and/or complex mental health disorders, and the need for teams to function interdependently [22]. Moreover, professionals whose performance is proficient, adaptive and proactive are more likely to implement evidence-based practices and to maintain greater fidelity to practice guidelines [24]. It is therefore important to identify variables associated with high work role performance in this context.

Certain models emanating from occupational psychology have been used in the health field for identifying relevant variables associated with team effectiveness and work role performance. For example, in the Integrated Team Effectiveness Model [15], external environments (social and policy context, organizational context) influence task-design variables (e.g. types and composition of teams, use of guidelines), which may be used by teams to improve effectiveness. According to Chiocchio [20], it is easier to predict work role performance than team effectiveness because behaviors are more heavily influenced by team processes and less by more distal variables from the external environments (e.g. mental health funding, network characteristics). The major determinants of work role performance involving team processes, as identified in the literature, include familiarity among team members [20], good communication [25], a high degree of collaboration and harmony [15], team interdependence [26], strong leadership [26], and trust [27]. By contrast, team effectiveness is hampered when confusion exists around the respective roles of team members [25, 28], conflicting ideologies and values, lack of mutual trust, and opposing views on user interests [25].

Studies have identified task-design variables associated with work role performance, such as team size, that may positively or negatively influence work role performance [15], as well as variables related to team diversity, which also have contradictory implications for work role performance [29, 30]. To the best of our knowledge, however, the effect of evidence-based practices (such as clinical approaches) on work role performance, or those of standardized procedures and evaluation tools, have never been assessed, despite the awareness that these variables improve quality of care for users with mental health and substance use disorders [10, 11]. Moreover, the influence of user characteristics on work role performance among teams has not been examined.

Relationships between work role performance and the external environments (including organizational context) have seldom been analyzed [15]. One exception is organizational culture, which was found to have an either positive or negative relationship with work role performance on teams [31–33]. Some studies found an association between work role performance and team effectiveness when integration strategies, such as interdisciplinary training [34, 35], were applied. Furthermore, no known studies have measured the association between work role performance and frequency of interaction with other teams or services. There is an assumption that frequent interaction with others professionals in mental health networks would increase trust, a variable strongly associated with work role performance [27].

Since task-design variables (e.g. standardized procedures, evaluation tools and user characteristics) and variables related to external environments (e.g. frequency and satisfaction of interactions with other teams or services) have rarely been examined in relation to work role performance, the present exploratory study aims to: 1) determine the association between work role performance and a wide range of variables related to team effectiveness gleaned from the literature, and 2) assess the covariance between each of these variables as well as their correlations with other exogenous variables. No known study has identified mediators of work role performance among mental health teams. Structural equation modelling was then used to define relationships between work role performance, variables associated with team effectiveness, and possible mediators.

## Materials and methods

### Study design and data collection

This study falls within a larger research project evaluating the implementation of mental health reforms [36] in Quebec (Canada) from 2005 to 2015 across 11 local health networks. The reforms sought to strengthen community mental health services by establishing primary care teams in 95 local health networks. It also promoted evidence-based practices and encouraged collaboration between primary care and specialized services through the implementation of several network integration strategies such as service agreements, liaison officers and shared training.

Networks were identified in consultation with 20 decision makers and selected according to the diversity of services offered, the integration strategies, the uptake of best practices and the level of implementation of the reforms. Two rural territories without specialized mental health services were removed from this study because they had too few interactions with other teams. Selected networks served populations of between 64,000 and 290,000 individuals.

An advisory committee with representatives from each selected network identified managers in each mental health team who could be recruited to the study. We collected data using structured, self-administered questionnaires with questions concerning mental health services in the previous 12 months. The questionnaires, adapted for primary care teams and specialized mental healthcare teams, were completed by managers (n = 88) between October 2013 and June 2014. They included 252 categorical and continuous items with five-point Likert scale responses. The mental health services questionnaires included the following elements: 1) manager characteristics (age, gender, seniority), 2) user characteristics (income, diagnosis), 3) team characteristics (types of primary care or specialized services, number and types of professionals), 4) clinical activities (frequency of use of standardized procedures and evaluation tools, and frequency of use of clinical approaches (e.g. cognitive behavior therapy), 5) organizational culture, 6) network integration strategies, 7) frequency and satisfaction regarding interactions with other teams and services, and 8) assessment of mental health services in the network. Team managers were encouraged to consult their organizational administrative data banks and their team members in completing the questionnaires. Completion of questionnaires required approximately one hour.

**Fig 1:**
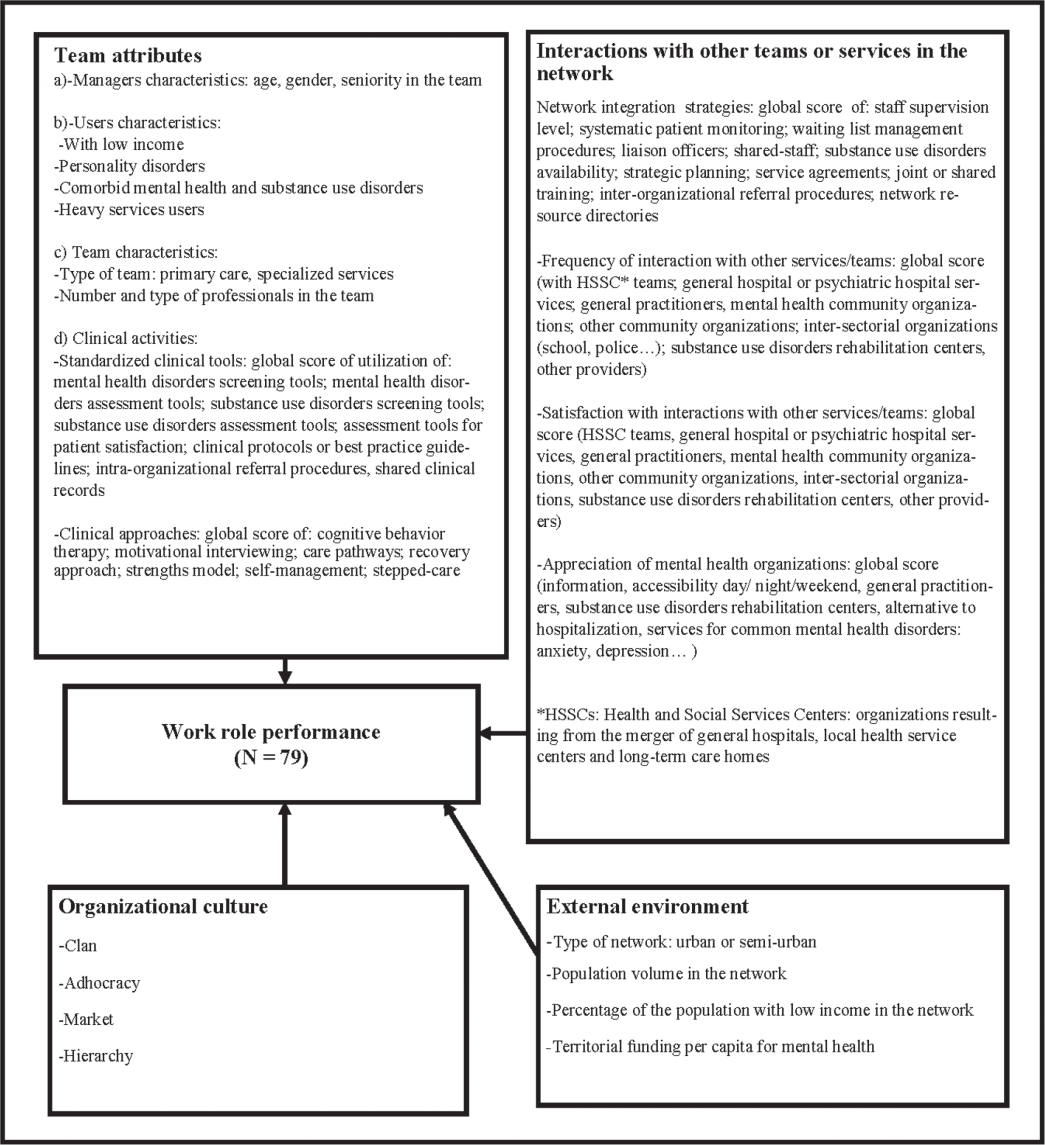
Conceptual framework. Independent Variables were grouped into four areas: 1) Team attributes; 2) Organizational culture; 3) Interactions with other teams or services in the network; and 4) External environment.

A conceptual framework (**Fig 1**) adapted from the Integrated Team Effectiveness Model [15] guided the analyses. The independent variables were organized from internal team-level characteristics to the external environment.

We measured the dependent variable “work role performance” with an adapted version of the Work Role Questionnaire [22]. Using a referent-shift approach [37], the nine original items designed to measure how individual behaviors among team members contribute to work role performance, for example: “I respond constructively to changes in the way my team works”, were adapted in order to solicit manager perceptions on how behaviors at the team-level may shape work role performance, for example: “Team members respond constructively to changes in the way my team works.” The aggregated Cronbach alpha (α) was 0.91, and ranged from 0.87 to 0.94 for each of the three sub-dimensions (proficiency, adaptivity, proactivity).

Organizational culture was assessed with the Organizational Culture Assessment Instrument (OCAI) [38]. This questionnaire covered six aspects. For each of them, participants had to distribute 100 points across four possible choices for each question. The OCAI identifies four organizational cultures: 1) Clan/Family, 2) Adhocracy/Entrepreneurial, 3) Market/Rational, and 4) Hierarchy/Bureaucratic [39].

Standardized procedures and evaluation tools related to clinical activities included MH disorder screening and assessment tools, substance use disorder screening and assessment tools, as well as other assesment tools for the following: patient satisfaction, clinical feedback procedures, clinical protocols, best practice guidelines, intra-organizational referral procedures, and shared clinical records. These variables were measured on five-point Likert scales (1 = almost never used; 5 = very often used) and merged into a global score (range from 9 to 45). As clinical approaches, it included cognitive behavior therapy, motivational interviewing, care pathways, recovery approach, strengths model, self-management, and stepped care. These variables were measured using five-point Likert scales (1 = almost never used; 5 = very often used) and then merged into a global score (range from 7 to 35). These variables, as well as a list of other key concepts included in this study, are described in an Appendix.

Network integration strategies included staff supervision level, systematic patient monitoring, waiting list management procedures, liaison officers, shared staff, service availability for substance use disorders, strategic planning, service agreements, shared training, inter-organizational referral procedures, and network resource directories. These variables were measured with a five-point Likert scale (1 = absent; 5 = completed implementation) and merged into a global score (range from 11 to 55) (see Appendix).

Finally, the frequency of interactions with other teams or services included interactions with primary care teams, general hospital or psychiatric hospital services, general practitioners, community mental health organizations (e.g. crisis centers, self-help groups), other community organizations (e.g. Alcoholics Anonymous, Narcotics Anonymous), inter-sectoral organizations (e.g. school, police), substance use disorder rehabilitation centers, and other providers (e.g. pharmacists). These variables were measured on a five-point Likert scale (1 = very low; 5 = very high), and also merged into a global score (range from 33 to 165).

All participants signed an informed consent form. The research ethics board of the Douglas Mental Health University Institute approved the multi-site study protocol (MP-IUMD-11037).

### Analyses

The data were scrutinized for missing values, univariate outliers, and normality assumptions (skewness and kurtosis). There was very little missing data, and no outliers. The pattern of missing data was analyzed with SPSS, 24^th^ edition, revealing a random distribution; the data were replaced by mean values. Variables not normally distributed were discarded in building the model. Default criteria for the model were not adjusted in order to achieve convergence. Univariate analyses comprised frequency distributions and percentages for categorical variables and central tendency measures for continuous variables (mean values and standard deviations). Variables included in the structural equation model (SEM) were those identified in the literature [10, 11, 40] related to team effectiveness. The purpose was to assess the relationships between exogenous and endogenous variables (work role performance scores) and also the magnitude of covariance among the exogenous variables.

The SEM of work role performance was computed using the program AMOS 21 for SPSS. It was built step by step guided by model quality parameters: chi-square statistics, degrees of freedom, and goodness-of-fit statistics. Regarding multivariate assumptions, a curve estimation testing all relationships between variables was calculated and found that they were sufficiently linear to be tested in the SEM model (P<0.000). Multicollinearity was also assessed using linear regression collinearity diagnostics: all variance inflator factors (VIF) were under 1.5, and all tolerance statistics were over 8.0. Direct and indirect effects, with and without mediators and bootstrapping, were estimated. Estimation and evaluation of fit were carried out using maximum likelihood tests.

The goodness-of-fit statistics relied notably on the root-mean-square residual (RMR), the comparative fit index (CFI), the root-mean-square error of approximation (RMSEA), the PCLOSE, and the Akaike information criterion (AIC). The p-value of the chi-square statistics reflects agreement between the SEM and the data. When the null hypothesis indicates that the SEM fits the data, the chi-square p-value should be non-significant (> 0.05) [41]. The RMR expresses the quotient by which the sample variances and co-variances differ from estimates; the smaller it is, the better the fit (0 meaning a perfect fit). The CFI constitutes the ratio between the model being estimated and the baseline model, based on discrepancy and degree of freedom[42]. A CFI close to 1 indicates an excellent fit. The RMSEA reflects the residuals in the model estimates. A RMSEA of 0.05 or less indicates a close fit [43]. The PCLOSE is the p-value from the assessment of the null hypothesis that the RMSEA does not exceed the alpha value of 0.05. A non-significant PCLOSE (> 0.05) indicates a close fit. The AIC is a comparative criterion of fit, which has no meaning without comparing different models. It reflects the balance between model fit and parsimony. The latter is violated when unnecessary parameters are retained in the model. As such, the AIC indicates improvement in model fit and parsimony from one model to another[44]. When comparing different models, smaller AIC indicates a better model.

## Results

Managers from 81 clinical teams out of 88 potential teams responded to the invitation to participate in this study. Two participants were excluded because their healthcare settings did not include specialized mental health services; the remaining 79 participants were recruited, representing a 92% response rate. Comparison analyses were made between the 79 participants and the seven1 non-participants with regard to the distributions for gender and type of healthcare settings (primary vs specialized healthcare services), and no significant differences were found (Gender: Pearson Chi-square = .604; df = 1; Fisher's Exact Test 2-sided P = .437; Type of healthcare setting: Pearson Chi-square = .604; df = 1; Fisher's Exact Test 2-sided P = .435). Among the 79 participating team managers, females outnumbered males by more than two to one (68% versus 32%). Their mean age was 44. Thirty-nine percent of managers came from primary healthcare services, and 61% from specialized healthcare services. Twenty-nine percent of managers (n = 23) worked in psychiatric hospitals and 71% (n = 56) in health and social service centers, which were institutions created by the merger of local community services centers, general hospitals and nursing homes under the Quebec reform. The most prevalent types of teams were inpatient units (n = 18; 23%), followed by out-patient clinics (n = 16; 20%), basic primary care mental health teams (n = 14; 18%), evaluation teams (n = 9; 11%), rehabilitation teams (n = 9: 11%), intensive case management teams (n = 8; 10%) and assertive community treatment teams (n = 5; 6%). Average seniority among team managers was six years. **Table 1** presents additional team characteristics. The overall work role performance score ranged from 23 to 45, with a mean of 36 (SD: 4.65).

**Table 1:** Team characteristics (N = 79).

		Minimum	Maximum	n/Mean	%/SD
Seniority within the team (in years)		0.	38	6.43	9.06
Healthcare work setting	Primary care service			31	39.2
	Specialized service			48	60.8
Frequency in use of standardized procedures and evaluation tools^a^		14.	37.	26.08	6.38
Frequency in use of clinical approaches^b^		8.	26.	18.38	3.74
Proportion of high users of mental health services within own clientele		0.	1	20.16	23.88
Proportion of users with personality disorders within own clientele		0	9	28.30	22.24
Frequency of interactions between own team and other professionals^c^		20	73	39.33	11.08
Implementation level for network integration strategies within own team^d^		16	40	28.15	6.60
Work role performance score^e^		23.	45	36.35	4.65

a Global score for the sum of all variables merged (n = 9; 1 to 5 for each variable); min: 9, max: 45; higher = more positive.

b Global score for the sum of all variables merged (n = 7; 1 to 5 for each variable); min: 7, max: 35; higher = more positive.

c Global score for the sum of all variables merged (n = 33; 1 to 5 for each variable); min: 33, max: 165; higher = more positive.

d Global score of the sum of all variables merged (n = 11; 1 to 5 for each variable); min: 11, max: 55; higher = more positive.

e Global score for the sum of all items (n = 9; 1 to 5 by item; min: 9, max: 45; higher = more positive.

**Fig 2:**
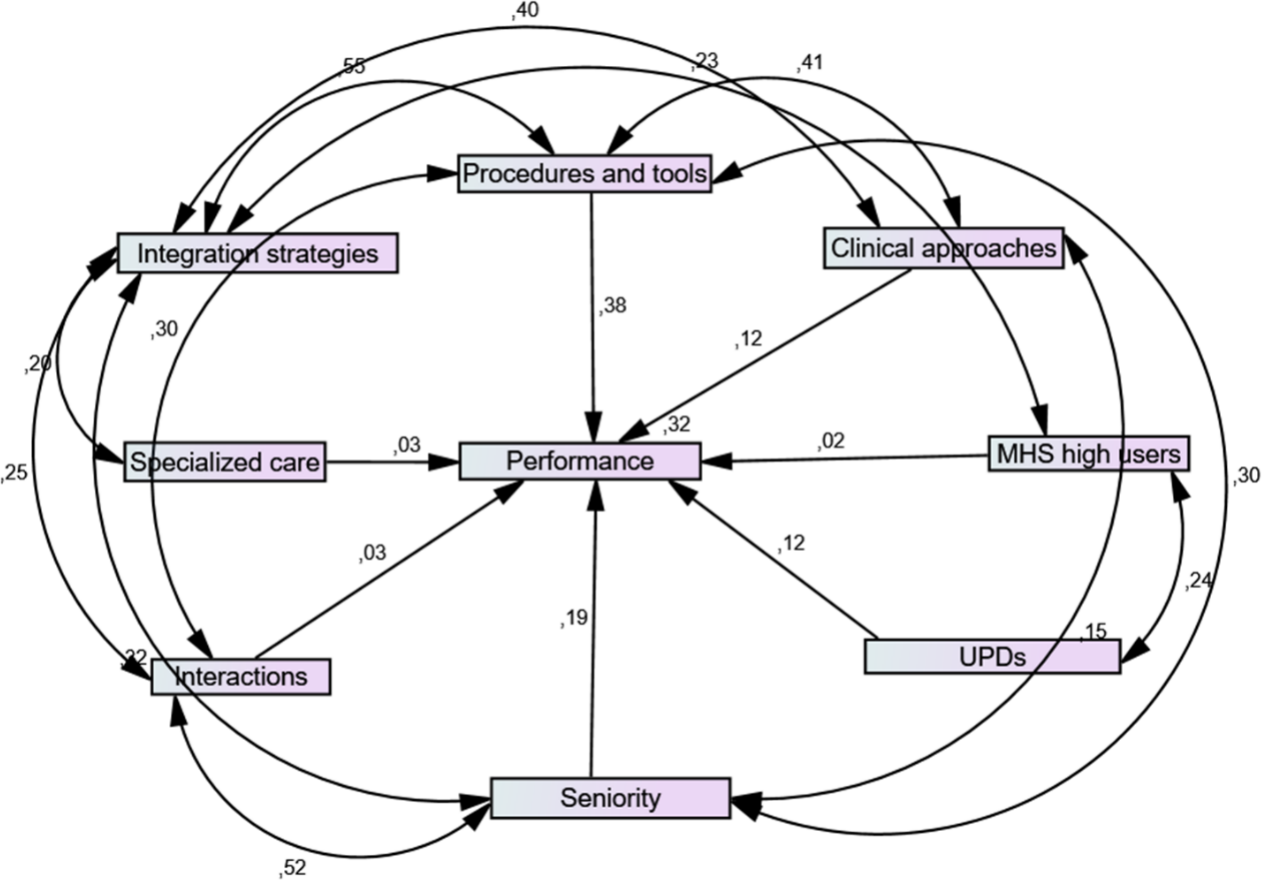
Structural equation model of relations between mental healthcare clinical team characteristics and work role performance. Single headed arrows show that work role performance is directly impacted by seven variables: clinical procedures and tools, clinical approaches, proportion of mental health service (MHS) high users, proportion of users with personality disorders (UPDs), seniority in the team, interactions with other MHS, and specialized care settings. Double headed arrows show covariances between some variables, notably interactions and seniority, integration strategies and clinical procedures and tools.

For the structural equation model (SEM: **Fig 2**), AMOS achieved a minimum chi-square of 0.022; df = 1; p = 0.883, meaning that the departure of the data from the model was non-significant. The goodness of fit resulted in the following estimates: RMR = 0.047; CFI: 1.000; RMSEA < 0.0001; PCLOSE: 0.894. These results indicated a nearly perfect model fit. For the standardized regression weights (**Table 2**), work role performance was significantly associated with frequency of use with respect to standardized procedures and evaluation tools (estimate: .0377; p = 0.001) and with seniority of the team manager (estimate: .0191; p = 0.044). Other variables were associated, although not significantly, with work role performance; they were, in decreasing order of importance: frequency of use of clinical approaches (clinical activities), proportion of users with personality disorders, proportion of high users of mental health services (user characteristics), specialized mental health services (team characteristics), and frequency of interactions with other teams or services.

**Table 2:** Standard regression weights of team characteristics for work role performance.

	Beta	95% confidence interval for beta		P value
		Lower	Upper	
Seniority of the team manager (in years)	0.191	0.034	0.396	0.044
Healthcare work setting: specialized mental health service	0.028	-0.124	0.206	0.694
Frequency of use of standardized procedures and evaluation tools	0.377	0.215	0.552	0.001
Frequency of use of clinical approaches	0.12	-0.035	0.254	0.195
Proportion of users with personality disorders within own clientele	0.115	-0.088	0.304	0.349
Proportion of high users of mental health services within own clientele	0.016	-0.14	0.155	0.869
Frequency of interaction between own team and other teams or services	0.032	-0.208	0.256	0.823

The highest estimates of covariance (**Table 3**) occurred between frequency of use of standardized procedures and evaluation tools, and the level of implementation for network integration strategies (estimate: 0.55; p = 0.001); between frequency of interactions with other teams or services and seniority of the team manager (estimate: 0.52; p = 0.001); between frequency of use of clinical approaches and frequency of use of standardized procedures and evaluation tools (estimate: 0.41; p = 0.001); and between frequency of use of clinical approaches and level of implementation of network integration strategies (estimate: 0.40; p = 0.001).

**Table 3:** Standardized estimates of covariance between exogenous variables related to work role performance.

Parameter			Estimate	P
Frequency of use of standardized procedures and evaluation tools	<-->	Level of implementation of network integration strategies within own team	0.55	0.001
Frequency of interactions between own team and other teams or services	<-->	Seniority of the team manager	0.52	0.001
Frequency of use of clinical approaches	<-->	Frequency of use of standardized procedures and evaluation tools	0.41	0.001
Frequency of use of clinical approaches	<-->	Level of implementation of network integration strategies within own team	0.40	0.001
Level of implementation of network integration strategies within own team	<-->	Seniority of the team manager	0.32	0.009
Frequency of interactions between own team and other teams or services	<-->	Frequency of use of standardized procedures and evaluation tools	0.30	0.035
Frequency of use of standardized procedures and evaluation tools	<-->	Seniority of the team manager	0.30	0.046
Frequency of interactions between own team and other teams or services	<-->	Level of implementation of network integration strategies within own team	0.25	0.044
Proportion of high users of mental health services within own clientele	<-->	Proportion of users with personality disorders within own clientele	0.24	0.019
Level of implementation of integration strategies within own team	<-->	Proportion of high users of mental health services within own clientele	0.19	0.028
Frequency of use of clinical approaches	<-->	Seniority of the team manager	0.15	0.026

## Discussion

The findings revealed that work role performance was associated almost exclusively with variables related to team attributes (procedures and tools, seniority in the team, clinical approaches, users with personality disorders, specialized care settings and high users of mental health services). The only other variables directly associated with work role performance was interaction with other teams or organizations. The most important finding relates to the close connection between work role performance and frequency of use of standardized procedures and evaluation tools. This association, which has yet to be identified in the literature, as far as we know, would seem perfectly reasonable. That is, the use of standardized procedures and evaluation tools has been found to increase proficiency and proactivity among mental health professionals [45], enabling them to better screen for mental disorders or substance use disorders [34] and to make informed decisions about treatment based on diagnosis. Considering the increasing frequency of complex mental health disorders and co-occurring mental disorders and substance use disorders [46], systematic use of these procedures and evaluation tools is essential in order to maintain proficiency among professionals [22]. Moreover, standardized procedures and evaluation tools facilitate development of a shared vision [47] which is essential for effective team integration [25] and work role performance. These procedures and tools also help to standardize care in multidisciplinary teams, improving quality of care, in turn [25].

The association between clinical approaches and work role performance also appears reasonable, as knowledge about multiple clinical approaches, and their use, allows mental health professionals to better adapt to various types of clientele and to address mental health issues, improving user outcomes [34]. These clinical approaches for mental health disorders (e.g. cognitive behavior therapy [48]) are generally considered evidence-based, as are other approaches geared to specific disorders such as depression (e.g. illness self-management [49]) or for substance use disorders (e.g. motivational interviewing [50]). Use of clinical approaches also involves sharing knowledge, skills, practices and experience among professionals on the same team and requires that they work together, improving communication and interpersonal relationships [40, 51].

The correlation between standardized procedures and evaluation tools, and clinical approaches also stands to reason, as these procedures and tools might be expected to help determine which clinical approaches should be used. There was also a high degree of covariance with network integration strategies in the study: standardized procedures, evaluation tools and clinical approaches are in fact included in the notion of network integration, and their use is known to produce better outcomes [52].

Another major finding concerned the link between work role performance and the seniority of team managers. Senior managers may be better acquainted with professionals, individually or collectively, and likely exercise greater leadership, which makes them more likely to influence team processes (e.g. coordination, knowledge-sharing, etc.) and performance [26]. According to the literature, the impact of leadership on individual and organizational performance is crucial [25, 53, 54]. The seniority of team managers also influences the frequency of interactions with other teams or services, support for network integration strategies and frequency of use among standardized procedures and evaluation tools. We may presume that experienced team managers would be more aware of existing resources, and would have formalized connections with them through various network integration strategies including service agreements, use of liaison officers, or joint training. Interaction with other teams or organizations may also familiarize team managers with new procedures, evaluation tools and clinical approaches that may be implemented within their teams.

Two user characteristics were associated with work role performance: proportion of users with personality disorders (UPDs) and proportion of high users. It is possible that these users require concerted action involving multiple health professionals; the strong correlation between these two user profiles is well documented in the literature. UPDs were identified as more likely to be high users of healthcare services, including primary care and specialized services [55–57]. A covariance emerged between network integration strategies and high users, but not between these strategies and UPDs. This seems to indicate that service integration focuses on users with complex problems [58] (e.g. co-occurring mental health and substance use disorders) rather than on patients with personality disorders only.

The association between specialized mental health teams and work role performance may reflect the longer-term establishment of specialized teams and their hierarchical structure, as compared with primary care teams. The more recent existence of the primary care teams in our sample also suggests that some may have lacked a full complement of staff, and that their team members were perhaps less well acquainted with each other. We also found a strong covariance between specialized mental health teams and use of network integration strategies. According to Liljegren [59], a higher degree of integration is required in order for specialization to occur. Providing continuity of care for users with severe mental health disorders after transfer to the community requires that institutions offering specialized services, such as psychiatric hospitals, formalize their interactions with other providers.

The association between frequent interactions with other teams and services, and work role performance further suggests that such interactions promote adaptivity and proactivity in mental health teams through the acquisition and mastery of new work methods or procedures. As mentioned previously, there was high covariance between frequency of interactions and the seniority of team managers, suggesting that experienced managers were more cognizant of existing resources. We also identified a strong link between specialized mental health teams and network integration strategies, which are designed precisely to ensure a closer working relationship among diverse teams and organizations. Indeed, specialized and primary care teams need to operate jointly if they are to offer the kind of biopsychosocial approach essential for effective treatment of people with a mental health condition [14].

Finally, the strong correlation between all variables associated with work role performance and network integration strategies, with the exception of proportion of UPDs, represented a major finding of this study. Inadequate treatment of complex or chronic mental health disorders often results from a disorganized healthcare system [60, 61]. Network integration strategies contribute to better quality, access and continuity of care [62], while helping prevent service fragmentation or duplication [63] and user dissatisfaction [64].

There are four important limitations to this study that should be noted. First, the data are cross-sectional and, as such, cannot be used to draw cause-and-effect conclusions. A longitudinal study may have revealed further differences in work role performance among mental health teams, both during and after the reform. Second, although our sample was sufficient to run the analyses, effects had to be substantial in order to be detected with only 79 participants. A larger sample would have provided more nuanced results. Third, only team managers were solicited for the study; the great majority responded to the questionnaires with assistance from their organizational administrative data banks and based on team consultations. Yet it is quite possible that their perceptions of work role performance may have differed from those of their team members. Variability in questionnaire responses among participating networks may also have been affected by whether or not managers actually carried out the consultations of administration data banks and their team members. Finally, the study design did not permit us to test for associations between work role performance and user-related outcomes, such as recovery, or quality of life.

## Conclusion

This exploratory study was innovative in assessing the perceptions of 79 mental health team managers in order to better understand the association between work role performance and a comprehensive range of variables related to team effectiveness, while also examining their inter-relationships with exogenous variables in the model. Work role performance in mental health teams was strongly influenced by the use of standardized procedures and evaluation tools and, to a lesser extent, by clinical approaches, as strategies that would also impact on quality of care for users with mental health disorders. The findings of our study suggest that healthcare systems should improve training in these areas. Most variables associated with work role performance were also linked with network integration strategies, suggesting that more systematic implementation of such strategies may indirectly increase work role performance to the ultimate benefit of users. Finally, the results confirm the influence of seniority among team managers on work role performance, but also on other associated variables, thus highlighting the value of stable management within healthcare systems.

## Supporting information

S1 File (DOCX)

## References

[1] Department of Health. No health without mental health: A cross-government mental health outcomes strategy of all ages England: National Health Service; 2011.

[2] Commonwealth of Australia. Fourth National Mental Health Plan- An agenda for collaborative government action in mental health 2009–2004: Commonwealth of Australia; 2009.

[3] Ministry of Social Affairs and Health. Plan for mental health and substance abuse work Proposal of the Mieli 2009 working group to develop mental and substance abuse work until 2015. Finland: Ministry of Social Affairs and Health; 2010.

[4] Nicaise P, Dubois V, Lorant V. Mental health care delivery system reform in Belgium: the challenge of achieving deinstitutionalisation whilst addressing fragmentation of care at the same time. Health Policy. 2014;115(2–3):120–7. doi: 10.1016/j.healthpol.2014.02.007 . 24582489 10.1016/j.healthpol.2014.02.007

[5] Rapp CA. The strength perspective of case management with persons suffering from severe mental illness In: Saleesbey D, editor. The strengths perspective in social work practice. New York: Longman; 1992 p. 45–58.

[6] Seekles W, van Straten A, Beekman A, van Marwijk H, Cuijpers P. Stepped care treatment for depression and anxiety in primary care. a randomized controlled trial. Trials. 2011;12:171 doi: 10.1186/1745-6215-12-171 ; PubMed Central PMCID: PMCPMC3152524. 21736720 10.1186/1745-6215-12-171 PMC3152524

[7] Hanrahan NP, Delaney K, Merwin E. Health care reform and the federal transformation initiatives: capitalizing on the potential of advanced practice psychiatric nurses. Policy Polit Nurs Pract. 2010;11(3):235–44. doi: 10.1177/1527154410390381 ; PubMed Central PMCID: PMCPMC3392024. 21233135 10.1177/1527154410390381 PMC3392024

[8] Jacob B, Macquet D, Natalis S. [A global reform of mental health care based on a community approach: the Belgian experience]. Sante Ment Que. 2014;39(1):209–42. . 25120123

[9] Kisely S, Lesage A. [Mental health services in Australia]. Sante Ment Que. 2014;39(1):195–208. . 25120122

[10] Peters AH, de Leeuw RJ, Schrijvers GJ. Integrating care for people with depression: developments in the Netherlands. Int J Integr Care. 2010;10:e62 ; PubMed Central PMCID: PMCPMC3031796. 21290001 10.5334/ijic.586 PMC3031796

[11] Franx G, Meeuwissen JA, Sinnema H, Spijker J, Huyser J, Wensing M, et al Quality improvement in depression care in the Netherlands: the Depression Breakthrough Collaborative. A quality improvement report. Int J Integr Care. 2009;9:e84 ; PubMed Central PMCID: PMCPMC2707591. 19590610 10.5334/ijic.314 PMC2707591

[12] Druss BG, von Esenwein SA. Improving general medical care for persons with mental and addictive disorders: systematic review. Gen Hosp Psychiatry. 2006;28(2):145–53. doi: 10.1016/j.genhosppsych.2005.10.006 . 16516065 10.1016/j.genhosppsych.2005.10.006

[13] van der Feltz-Cornelis CM. Ten years of integrated care for mental disorders in the Netherlands. Int J Integr Care. 2011;11 Spec Ed:e015 ; PubMed Central PMCID: PMCPMC3111888. 21677846 10.5334/ijic.567 PMC3111888

[14] Harris M, Greaves F, Gunn L, Patterson S, Greenfield G, Car J, et al Multidisciplinary integration in the context of integrated care—results from the North West London Integrated Care Pilot. Int J Integr Care. 2013;13:e041 ; PubMed Central PMCID: PMCPMC3817952. 24198737 10.5334/ijic.1146 PMC3817952

[15] Lemieux-Charles L, McGuire WL. What do we know about health care team effectiveness? A review of the literature. Med Care Res Rev. 2006;63(3):263–300. doi: 10.1177/1077558706287003 . 16651394 10.1177/1077558706287003

[16] Quaschning K, Korner M, Wirtz M. Analyzing the effects of shared decision-making, empathy and team interaction on patient satisfaction and treatment acceptance in medical rehabilitation using a structural equation modeling approach. Patient Educ Couns. 2013;91(2):167–75. doi: 10.1016/j.pec.2012.12.007 . 23318156 10.1016/j.pec.2012.12.007

[17] Wagner EH. The role of patient care teams in chronic disease management. BMJ. 2000;320(7234):569–72. ; PubMed Central PMCID: PMCPMC1117605. 10688568 10.1136/bmj.320.7234.569 PMC1117605

[18] Grumbach K, Bodenheimer T. Can health care teams improve primary care practice? JAMA. 2004;291(10):1246–51. doi: 10.1001/jama.291.10.1246 . 15010447 10.1001/jama.291.10.1246

[19] Kilpatrick K, Lavoie-Tremblay M, Ritchie JA, Lamothe L. Advanced practice nursing, health care teams, and perceptions of team effectiveness. Health Care Manag (Frederick). 2011;30(3):215–26. doi: 10.1097/HCM.0b013e318225e03a . 21808173 10.1097/HCM.0b013e318225e03a

[20] Chiocchio F. Indicateurs pertinents à la collaboration dans le milieu de la santé - cadre conceptuel et inventaire de mesures Québec, Canada: Université de Montréal, 2012.

[21] Salas E, Burke CS, Cannon-Bowers JA. Teamwork: emerging principles. Int J Manag Rev. 2000;2(4):339–56. 0.1111/1468-2370.00046.

[22] Griffin MA, Neal A, Parker SK. A new model of work role performance: positive behavior in uncertain and interdependent contexts. Acad Manag J. 2007;50(2):327–47.

[23] Frese M, Fay D. Personal initiative: An active performance concept for work in the 21st century. Res Organ Behav. 2001;23.

[24] Mowbray CT, Holter MC, Teage GB, Bybee D. Fidelity Criteria: Development, Measurement, and Validation. Am J Eval. 2003;24(3):315–40.

[25] Suter E, Oelke ND, Adair CE, Armitage GD. Ten key principles for successful health systems integration. Healthc Q. 2009;13 Spec No:16–23. ; PubMed Central PMCID: PMC3004930. 20057244 10.12927/hcq.2009.21092 PMC3004930

[26] Mathieu J, Maynard MT, Rapp T, Gilson L. Team Effectiveness 1997–2007: A review of recent advancements and a glimpse into the future. J Manag. 2008;34:410–77.

[27] Chiocchio F, Forgues D, Paradis D, Iordanova A. Teamwork in Integrated Design Projects: Understanding the Effects of Trust, Conflict, and Collaboration on Performance. PMJ. 2011;42(6):78–91.

[28] Maslin-Prothero SE, Bennion AE. Integrated team working: a literature review. Int J Integr Care. 2010;10:e043 ; PubMed Central PMCID: PMCPMC2883237. 20543972 PMC2883237

[29] Kozlowski SWJ, Bell B. Work groups and teams in organizations In: Weiner IB, Schmidt NW, Highouse S, editors. Handbook of Psychology Industrial and Organizational Psychology. 12 London: Wiley; 2003 p. 333–75.

[30] Blackmore G, Persaud DD. Diagnosing and improving functioning in interdisciplinary health care teams. Health Care Manag (Frederick). 2012;31(3):195–207. doi: 10.1097/HCM.0b013e3182619d48 . 22842755 10.1097/HCM.0b013e3182619d48

[31] Heritage B, Pollock C, Roberts L. Validation of the organizational culture assessment instrument. PLoS One. 2014;9(3):e92879 doi: 10.1371/journal.pone.0092879 ; PubMed Central PMCID: PMC3965488. 24667839 10.1371/journal.pone.0092879 PMC3965488

[32] Scott T, Mannion R, Marshall M, Davies H. Does organisational culture influence health care performance? A review of the evidence. Journal of health services research & policy. 2003;8(2):105–17. doi: 10.1258/135581903321466085 . 12820673 10.1258/135581903321466085

[33] Gerowitz MB, Lemieux-Charles L, Heginbothan C, Johnson B. Top management culture and performance in Canadian, UK and US hospitals. Health Serv Manage Res. 1996;9(2):69–78. doi: 10.1177/095148489600900201 . 10156914 10.1177/095148489600900201

[34] Buljac-Samardzic M, Dekker-van Doorn CM, van Wijngaarden JD, van Wijk KP. Interventions to improve team effectiveness: a systematic review. Health Policy. 2010;94(3):183–95. doi: 10.1016/j.healthpol.2009.09.015 . 19857910 10.1016/j.healthpol.2009.09.015

[35] McEwan D, Ruissen GR, Eys MA, Zumbo BD, Beauchamp MR. The Effectiveness of Teamwork Training on Teamwork Behaviors and Team Performance: A Systematic Review and Meta-Analysis of Controlled Interventions. PLoS One. 2017;12(1):e0169604 doi: 10.1371/journal.pone.0169604 ; PubMed Central PMCID: PMCPMC5234826. 28085922 10.1371/journal.pone.0169604 PMC5234826

[36] MSSS. Plan d'action en santé mentale 2005–2010—La force des liens Québec: Ministère de la Santé et des Services sociaux 2005.

[37] Chan D. Functional relations among constructs in the same content domain at different levels of analysis: A typology of composition models. J Appl Psychol. 1998;83(2):234–46.

[38] Cameron KS, Quinn RE. Diagnosing and changing organizational culture based on the Competing Values Framework. Revised edition San Fransisco: Jossey-Bass; 2006.

[39] Scammon DL, Tabler J, Brunisholz K, Gren LH, Kim J, Tomoaia-Cotisel A, et al Organizational culture associated with provider satisfaction. J Am Board Fam Med. 2014;27(2):219–28. doi: 10.3122/jabfm.2014.02.120338 ; PubMed Central PMCID: PMC4097883. 24610184 10.3122/jabfm.2014.02.120338 PMC4097883

[40] Fleury MJ. Integrated service networks: the Quebec case. Health Serv Manage Res. 2006;19(3):153–65. doi: 10.1258/095148406777888080 . 16848956 10.1258/095148406777888080

[41] Bollen KA. Structural equations with latent variables New York: Wiley; 1989.

[42] Bentler PM. Comparative fit indexes in structural models. Psychol Bull. 1990;107(2):238–46. . 2320703 10.1037/0033-2909.107.2.238

[43] Browne MW, Cudeck R. Alternative ways of assessing model fit In: Bollen KA, Long JSE, editors. Testing structural equation models. Newbury Park, CA: Sage; 1993 p. 136–62.

[44] Akaike H. Factor analysis and AIC. Psychometrika. 1987;52(3):317–22.

[45] Lyon AR, Dorsey S, Pullmann M, Silbaugh-Cowdin J, Berliner L. Clinician use of standardized assessments following a common elements psychotherapy training and consultation program. Adm Policy Ment Health. 2015;42(1):47–60. doi: 10.1007/s10488-014-0543-7 ; PubMed Central PMCID: PMCPMC4155022. 24590606 10.1007/s10488-014-0543-7 PMC4155022

[46] Kessler RC. The epidemiology of dual diagnosis. Biol Psychiatry. 2004;56(10):730–7. doi: 10.1016/j.biopsych.2004.06.034 . 15556117 10.1016/j.biopsych.2004.06.034

[47] Fleury MJ, Perreault M, Grenier G, Imboua A, Brochu S. Implementing Key Strategies for Successful Network Integration in the Quebec Substance-Use Disorders Programme. Int J Integr Care. 2016;16(1):7 doi: 10.5334/ijic.2457 ; PubMed Central PMCID: PMCPMC5015544. 27616951 10.5334/ijic.2457 PMC5015544

[48] Beck JS. Cognitive Behavior Therapy: Basics and beyond. 2nd ed New York: Guilford Press; 2011.

[49] Miller WR. Motivational interviewing: research, practice and puzzles. Addict Behav. 1996;21(6):835–42. 8904947 10.1016/0306-4603(96)00044-5

[50] Bilsker D, Goldner EM, Anderson E. Supported self-management: a simple, effective way to improve depression care. Can J Psychiatry. 2012;57(4):203–9. doi: 10.1177/070674371205700402 . 22480584 10.1177/070674371205700402

[51] Janse B, Fabbricotti IN, Huijsman R. The effects of integrated care on professionals: a systematic review. Int J Integr Care. 12(Suppl 3):e 184.

[52] Fleury MJ, Perreault M, Grenier G, Imboua A, Brochu AB. Implementing Key Strategies for Successful Network Integration in the Quebec Substance-Use Disorders Programme. Int J Integr Care. 2016;16(1):7 doi: 10.5334/ijic.2457 27616951 10.5334/ijic.2457 PMC5015544

[53] de Stampa M, Vedel I, Mauriat C, Bagaragaza E, Routelous C, Bergman H, et al Diagnostic study, design and implementation of an integrated model of care in France: a bottom-up process with continuous leadership. Int J Integr Care. 2010;10:e034 ; PubMed Central PMCID: PMC2834925. 20216954 PMC2834925

[54] Miller JL. A post-mortem on healthcare integration: an organizational development approach. Healthc Leadersh Manag Rep. 2000;8(9):5–15. . 11184824

[55] Huynh C, Ngamini Ngui A, Kairouz S, Lesage A, Fleury MJ. Factors associated with high use of general practitioner and psychiatrist services among patients attending an addiction rehabilitation center. BMC Psychiatry. 2016;16:258 doi: 10.1186/s12888-016-0974-7 ; PubMed Central PMCID: PMCPMC4957405. 27450676 10.1186/s12888-016-0974-7 PMC4957405

[56] Goodman M, Patil U, Steffel L, Avedon J, Sasso S, Triebwasser J, et al Treatment utilization by gender in patients with borderline personality disorder. J Psychiatr Pract. 2010;16(3):155–63. doi: 10.1097/01.pra.0000375711.47337.27 . 20485103 10.1097/01.pra.0000375711.47337.27

[57] Sansone RA, Farukhi S, Wiederman MW. Utilization of primary care physicians in borderline personality. Gen Hosp Psychiatry. 2011;33(4):343–6. doi: 10.1016/j.genhosppsych.2011.04.006 . 21762830 10.1016/j.genhosppsych.2011.04.006

[58] Kodner DL. All together now: a conceptual exploration of integrated care. Healthc Q. 2009;13 Spec No:6–15. . 20057243 10.12927/hcq.2009.21091

[59] Liljegren A. Strategic collaboration councils in the mental health services: what are they working with? Int J Integr Care. 2013;13:e009 Epub 2013/05/21. ; PubMed Central PMCID: PMC3653287. 23687481 10.5334/ijic.838 PMC3653287

[60] Sampalli T, Fox RA, Dickson R, Fox J. Proposed model of integrated care to improve health outcomes for individuals with multimorbidities. Patient preference and adherence. 2012;6:757–64. doi: 10.2147/PPA.S35201 ; PubMed Central PMCID: PMCPMC3484525. 23118532 10.2147/PPA.S35201 PMC3484525

[61] Valentijn PP, Schepman SM, Opheij W, Bruijnzeels MA. Understanding integrated care: a comprehensive conceptual framework based on the integrative functions of primary care. Int J Integr Care. 2013;13:e010 ; PubMed Central PMCID: PMCPMC3653278. 23687482 10.5334/ijic.886 PMC3653278

[62] Holmesland AL, Seikkula J, Nilsen O, Hopfenbeck M, Erik Arnkil T. Open Dialogues in social networks: professional identity and transdisciplinary collaboration. Int J Integr Care. 2010;10 ; PubMed Central PMCID: PMCPMC2948679. 20922064 10.5334/ijic.564 PMC2948679

[63] Ye C, Browne G, Grdisa VS, Beyene J, Thabane L. Measuring the degree of integration for an integrated service network. Int J Integr Care. 2012;12:e137 ; PubMed Central PMCID: PMCPMC3601536. 23593050 10.5334/ijic.835 PMC3601536

[64] Macadam M. Progress toward integrating care for seniors in Canada: "We have to skate toward where the puck is going to be, not to where it has been.". Int J Integr Care. 2011;11 Spec Ed:e016 ; PubMed Central PMCID: PMCPMC3111885. 21677847 PMC3111885

